# A Systematic Review of the Comparative Efficacy of Lactobacillus Probiotics and Sodium Hypochlorite as Intracanal Irrigants Against Enterococcus faecalis

**DOI:** 10.7759/cureus.70926

**Published:** 2024-10-06

**Authors:** Mrinalini Mrinalini, Alpa Gupta, Dax Abraham, Arun Kumar Duraisamy, Rajat Sharma

**Affiliations:** 1 Department of Conservative Dentistry and Endodontics, Manav Rachna Dental College, Faridabad, IND

**Keywords:** e. faecalis, irrigant, lactobacillus, probiotics, sodium hypochlorite

## Abstract

Irrigation plays a pivotal role in the success of root canal treatments. The development of innovative, less hazardous irrigating solutions is necessary because of the inherent limitations of the gold standard, sodium hypochlorite. Since probiotics have proven to be effective in treating common oral diseases such as periodontitis and dental caries, they have gained attention in the field of endodontics as well. The present systematic review aims to assess the efficacy of Lactobacillus probiotics against *Enterococcus faecalis* compared to that of sodium hypochlorite. A thorough search of five databases, PubMed, Scopus, EBSCOhost, ScienceDirect, and BVS (Biblioteca Virtual en Salud, or Virtual Health Library), from January 2000 to January 2024 yielded 135 articles after a preliminary search. Three research publications that satisfied the strict inclusion and exclusion criteria were charted after the removal of duplicates and careful examination of the full-text articles. The Quality Assessment Tool for In Vitro Studies (QUIN Tool) was used to assess the quality of the study, aiming to identify any risk of bias. All three publications had a low risk of bias and demonstrated that Lactobacillus species were effective against *E. faecalis* and proved to be a safer alternative to sodium hypochlorite as an intracanal irrigant. However, more clinical trials are required to determine the best probiotic combinations, appropriate probiotic carriers, and ideal dosage and frequency of administration before using probiotics as intracanal irrigants in clinical trials.

## Introduction and background

Probiotics are live microorganisms that provide health benefits to the host when taken in adequate amounts [1]. Lilly and Stillwell were the first to use the term “probiotic” in 1965, as opposed to the term “antibiotic” [2]. Hull's groundbreaking study from 1984 established *Lactobacillus acidophilus* as the initial probiotic species [3]. In 1994, the World Health Organization declared probiotics to be an essential immune defense system. This was crucial due to the rising issue of antibiotic resistance [4]. To date, 13 probiotics have been evaluated in dentistry and medicine, showing promising results that prompted further research in this field.

The primary objective of managing an infected root canal system is to eliminate the key etiological factors through chemomechanical preparation. Root canals are enlarged using various instruments to facilitate thorough cleaning and shaping. Sodium hypochlorite stands out as the predominant irrigant to further reduce bacterial load and is considered the gold standard in root canal irrigants [5].

*Enterococcus* *faecalis* exhibits notable resistance to commonly employed medicaments and irrigants, owing to its proton pump [6]. Its survival after obturation is often a concern given its capacity for independent living and resistance to antibacterial treatments [7]. Furthermore, the biofilm formed by *E. faecalis *aids in its defense against systemic interventions and host defense.

Given the increasing isolation of *E. faecalis* in failed root canal cases, it has garnered significant attention within dental communities [8]. Various approaches to combat *E. faecalis* have been explored. However, with the increasing demand for a more holistic approach to treatment, bacteriotherapy was introduced in the field of endodontics. A preliminary study conducted by Bohara and Kokate in 2017 to evaluate the role of probiotics against endodontic pathogens concluded the efficacy of Lactobacillus and Bifidobacterium probiotics against endodontic pathogens [9].

Probiotics are live microorganisms that confer health benefits to the host when administered in an adequate amount [10]. They have been investigated for their potential in treating oral health issues, particularly in addressing periodontal problems, halitosis, and preventing cavities [11,12]. Recently, this bacteriotherapy has gained much attention in the field of endodontics. The proposed mechanisms underlying the efficacy of probiotics encompass diverse actions, such as impeding the formation of pathogenic biofilms, eliciting the synthesis of cytoprotective proteins, reduction of inflammation, activation of the host immune system, eradicating pathogens through the production of bacteriocins, acids, and peroxides, and modulating the local pH environment [13].

The present review aims to comprehensively synthesize the latest findings related to the assessment of effectiveness of Lactobacillus probiotics against *E. faecalis* in comparison with sodium hypochlorite.

## Review

Methods

Literature Search Strategy

The systematic review methodology was formulated by employing the most recent Preferred Reporting Items for Systematic Reviews and Meta-Analyses (PRISMA) checklist. The Population, Intervention, Comparison, Outcome, and Study Design (PICOS) criteria were considered while conducting the systematic review. It was further registered in the Open Science Framework (https://osf.io/). The primary research question was whether Lactobacillus species probiotics demonstrate better efficacy as intracanal irrigants compared to sodium hypochlorite against *E. faecalis*. The PICOS framework for the eligibility criteria included the following components: Population (P): *E. faecalis*; Intervention (I): Lactobacillus probiotics; Control (C): sodium hypochlorite; outcome (O): reduction or elimination of *E. faecalis*; Study design (S): in vitro and ex vivo studies.

The relevant literature search involved searches across five databases, PubMed, Scopus, EBSCOhost, ScienceDirect, and BVS (Biblioteca Virtual en Salud, or Virtual Health Library), from January 2000 to January 2024. MeSH terms were employed, and the search queries were constructed using Boolean operators ("AND" and "OR"). The search criteria were as follows: (("Lactobacillus"[MeSH Terms] OR "Lactobacillus"[All Fields]) AND "E.faecalis"[All Fields] AND ("sodium hypochlorite"[MeSH Terms] OR ("sodium"[All Fields] AND "hypochlorite"[All Fields]) OR "sodium hypochlorite"[All Fields])) AND (english[Filter]). Three examiners scrutinized the search database, and inclusion/exclusion decisions were based on specific criteria.

Inclusion and Exclusion Criteria

The inclusion criteria for this study comprised several key aspects. First, only studies published in English were included. Additionally, eligible studies had to be original research focused on evaluating the efficacy of Lactobacillus probiotics against *E. faecalis*. Furthermore, the included studies needed to compare the Lactobacillus species as probiotics with sodium hypochlorite.

Conversely, studies involving probiotics other than Lactobacillus species were excluded. Research comparing the effectiveness of Lactobacillus species with any other irrigant besides sodium hypochlorite against *E. faecalis* was also excluded, irrespective of the concentration used. Additionally, studies that evaluated probiotics and sodium hypochlorite against bacteria other than *E. faecalis* were excluded. Language restrictions were strictly adhered to, with only English-language publications being included. Editorials, commentaries, and case reports were excluded from this review.

Critical Appraisal/Selection of Studies

In the initial phase, duplicated studies were excluded, ensuring that each study was considered only once. The titles and abstracts of the chosen studies were then scrutinized independently by two authors (MM and AG). In cases where a definitive judgment could not be made based on the title and abstract alone, the full texts were thoroughly examined for the ultimate decision. In the subsequent phase, the full texts of potentially eligible studies were carefully reviewed using the PICOS strategy and assessed against the eligibility criteria. Any disagreements regarding the inclusion of studies were resolved by consensus with a third author (DA). The agreement among the authors was confirmed using the Cohen kappa test (>0.8).

Data Extraction

Data from the included studies were independently gathered by two authors (MM and AG). Any discrepancies were resolved through consultation with a third author (DA). The extracted information encompassed details such as author(s), year of publication, type of study, sample size, species of Lactobacillus used, concentration of sodium hypochlorite, and method of evaluation of the outcome. The studies selected were tabulated and further subjected to the inclusion and exclusion criteria.

Evaluation of Methodological Quality

During the data extraction phase, two review authors (MM and AG) appraised the risk of bias (RoB) inherent in the included studies.

Results

**Figure 1:**
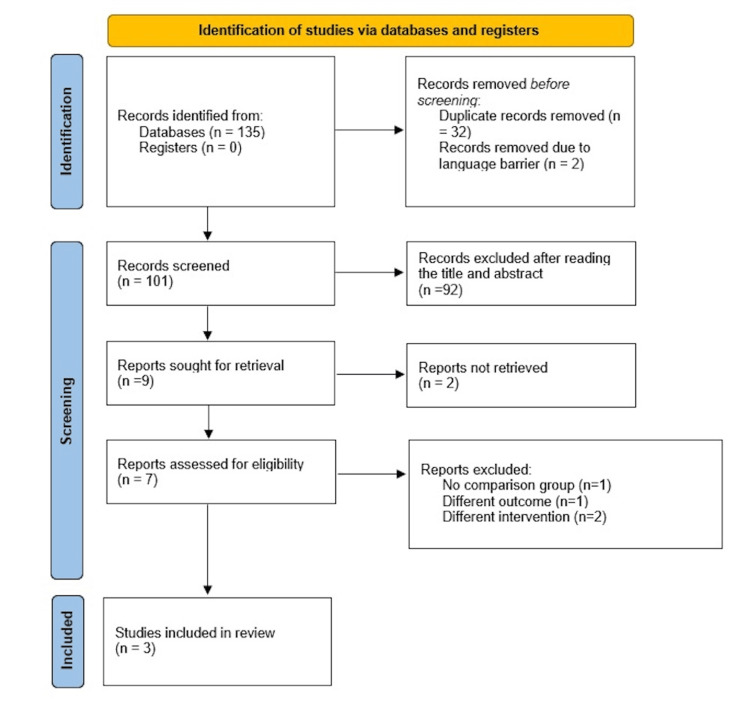
Preferred Reporting Items for Systematic Reviews and Meta-Analyses (PRISMA) flow diagram for study selection

A preliminary exploration of the database yielded 135 articles, and the subsequent removal of duplicates resulted in 103 unique articles. Two studies were omitted due to language barriers. Upon scrutinizing the titles and abstracts of the remaining 101 articles and their cross-references, nine articles were selected. The full texts of two articles could not be retrieved, and a further examination of the full texts of the remaining seven articles led to the exclusion of four articles that did not align with the predefined inclusion criteria. The comprehensive selection process and inclusion of articles are visually represented in Figure 1 through the PRISMA flow diagram.

Study Characteristics

The data collected from the three included studies are summarized in Table 1.

**Table 1:** Data collected from the included studies

Title	Author, year	Type of study	Sample size/population	Experimental group (Lactobacillus species used)	Control group (sodium hypochlorite concentration)	Method of evaluation of outcome	Outcome
The products of probiotic bacteria effectively treat persistent Enterococcus faecalis biofilms	Safadi et al., 2022 [14]	In vitro	-	*Lactobacillus casei*, *Lactobacillus plantarum*	1%, 3%, and 5%	Growth measurement using optical density	Sodium hypochlorite irrigations may contribute to the persistence of biofilm cells if used at concentrations lower than 3%. Probiotic strains and their products represent a new reservoir of biofilm therapies for *E. faecalis* infections formed in the root canal system.
A promising probiotic irrigant: an in vitro study	El-Sayed et al., 2019 [15]	In vitro	N=14	*Lactobacillus rhamnosus* (*L. rhamnosus* B-445)	2.5%	Colony count	The previous results revealed that the NaOCl irrigant group had the lowest mean value of (log 10 CFU/ml) of *E. faecalis* after immediate irrigation (0.00) and after 24 hours post-irrigation (2.20 ± 2.01) followed by the probiotic group with a mean value of 4.95 ± 0.11 for immediate irrigation and 4.40 ± 0.10 for post-irrigation.
The potential of reuterin derived from Indonesian strain of *Lactobacillus reuteri *against endodontic pathogen biofilms in vitro and ex vivo	Widyarman et al., 2023 [16]	In vitro and ex vivo	N=20	*Lactobacillus reuter*i LC 382 415 and *Lactobacillus reuteri* Prodentis derivative	2.5%	Time-dependent assay at 5 min and 30 min after irrigation by colony-forming units assay, confirmed using real-time PCR	Reuterin isolated from *L. reuteri *has the ability to inhibit in vitro and ex vivo biofilms of endodontic pathogens, such as *E. faecalis*.

Study Type

All three of the included studies were conducted in vitro. Notably, the third study by Widyarman et al. featured a bifurcated approach, with the latter stage transitioning to an ex vivo environment [16]. The study by El-Sayed et al. appraised the direct impact of Lactobacillus probiotics against the pathogen, while the remaining two studies focused on the utilization of probiotic-secreted products [15]. Additionally, the two studies assessed the efficacy of probiotics against pathogenic biofilm formation, whereas the study done by El-Sayed et al. evaluated their effectiveness in the planktonic state [14-16].

Probiotics Used and Their Administration

The different species of Lactobacillus used were *Lactobacillus plantarum *and *Lactobacillus casei* (used in the study by Safadi et al. [14]), *Lactobacillus rhamnosus* (used in the study by El-Sayed et al. [15]), and *Lactobacillus reuteri* (used in the study by Widyarman et al. [16]).

El-Sayed and colleagues utilized lyophilized *Lactobacillus rhamnosus* B-445 (Northern Regional Research Laboratory, Illinois, USA) to create an experimental irrigant. They prepared the irrigant by introducing 5 ml of the probiotic (2 x 10^8^ CFU/ml) into 10 ml of sterile distal water. The irrigation was administered using a 30-gauge side-vented needle, with 5 ml of the irrigant delivered [15].

Widyarman et al. employed *Lactobacillus reuteri* LC 382 415 and *Lactobacillus reuteri *Prodentis (BioGaia, Stockholm, Sweden) as the subject of investigation. This bacterial strain is recognized for its production of various antimicrobial compounds, including lactic acid, ethanol, and reuterin, which collectively contribute to its efficacy against pathogens. The isolation of reuterin was accomplished through the centrifugation of the bacterial suspension, followed by the filtration of the resulting supernatants using a 0.2-micrometer filter; 5 ml of the irrigant was delivered over a period of five minutes and the depth of the irrigating needle was kept at two-third of the root length. A concentration of 100 µg/ml was found to be the most effective [16].

Control Group

Safadi et al. used different concentrations of sodium hypochlorite (1%, 3%, and 5%) and concluded that when sodium hypochlorite is used at concentrations less than 3%, it results in the persistence of biofilm. Though sodium hypochlorite was able to completely eradicate the planktonic form of bacteria, its efficacy was reduced drastically in the biofilm sample [14]. El-Sayed et al. used 2.5% sodium hypochlorite, as the control group, and showed that the lowest mean value of *E. faecalis* was seen immediately as well as 24-hour post-irrigation when compared to the probiotics or saline group [15]. Widyarman et al. used sodium hypochlorite at a concentration of 2.5% [16].

Method of Evaluation

The effect of probiotics and sodium hypochlorite was evaluated against *E. faecalis *through growth measurement using optical density by Safadi et al., colony count by El-Sayed et al., and colony-forming assay and PCR by Widyarman et al. [14-16].

Despite the exclusion of certain studies in this review, substantial heterogeneity was observed, preventing the implementation of a meta-analysis. Consequently, a comprehensive analysis of the retained studies was conducted.

Study Quality Assessment

The Quality Assessment Tool for In Vitro Studies (QUIN Tool) was utilized to conduct a quality assessment of the studies to determine the risk of bias. Evaluations for RoB included the goals and objectives, sample calculation, sampling procedure, group details, methodology explanation, operator details, randomization, outcome assessment method, outcome assessor details, blinding, statistical analysis, and result presentation. A sign of “X” for not specified, “-” for inadequately specified, or “+” for adequately specified was assigned to each parameter. Studies that did not report one to three of the items were classified as low-risk studies, those that did not report four to six items were considered as moderate-bias studies, and studies with more than six non-reported items were considered as having a high risk of bias. All the three studies included had a low risk of bias (Table 2).

**Table 2:** Risk of bias of included studies X: not specified; +: adequately specified

	Clearly stated aims and objectives	Detailed explanation of sample size calculation	Detailed explanation of the sampling technique	Details of the comparison group	Detailed explanation of methodology	Operator details	Randomization	Method of measurement of outcome	Outcome assessor details	Blinding	Statistical analysis	Presentation of results	Overall assessment
Safadi et al., 2022 [14]	+	X	X	+	+	X	+	+	+	+	+	+	Low risk of bias
El-Sayed et al., 2019 [15]	+	X	X	+	+	X	+	+	+	+	+	+	Low risk of bias
Widyarman et al., 2023 [16]	+	X	X	+	+	X	+	+	+	+	+	+	Low risk of bias

Discussion

The efficacy of endodontic treatment is significantly influenced by the disinfection process. An ideal root canal irrigation protocol should not only eliminate bacteria, biofilm, and smear layer but also disinfect anatomical complexities. Sodium hypochlorite is the most frequently employed irrigant owing to its antimicrobial efficacy, capacity to dissolve organic matter, and cost-effectiveness [17]. However, drawbacks associated with sodium hypochlorite include its pronounced toxicity upon accidental periradicular tissue injection, unpleasant odour and taste, and the potential to bleach clothing and corrode metallic objects [18]. Furthermore, sodium hypochlorite significantly influences the mechanical properties of dentin, encompassing microhardness, roughness, elastic modulus, flexural strength, inorganic content, and the organic-inorganic ratio [19,20]. A randomized controlled trial conducted by Verma in 2019 reported the ineffectiveness of 1.3% and 2.5% sodium hypochlorite concentrations in removing bacterial loads [21]. Additionally, hypochlorite proves inadequate in eliminating the inorganic constituents of the smear layer and the hard-tissue debris that accrues during mechanical instrumentation [22]. In the studies included in the present systematic review, El-Sayed et al. and Widyarman et al. used 2.5% sodium hypochlorite whereas Safadi et al. used 1%, 3%, and 5% sodium hypochlorite [14-16].

Given the aforementioned limitations of sodium hypochlorite, exploration for alternative irrigants has been undertaken. A more holistic approach to bacteriotherapy, inspired by its successful outcomes in systemic disease management, has recently gained considerable attention. Subsequently, these probiotics have demonstrated significant effectiveness in the management of dental caries and periodontitis. This prompted the initiation of trials to assess their application in endodontics as well.

Outcome

El-Sayed et al. concluded that* Lactobacillus rhamnosus* can inhibit the growth of *E. faecalis* and shows potential as a novel safe irrigant in endodontics [15]. Gupta supported the efficacy of probiotics in pathogen elimination, citing mechanisms like the production of bacteriocin-like inhibitory substances (BLIS) and alteration of local environmental pH [23]. The probiotic irrigant resulted in more *Lactobacillus rhamnosus* colonies than *E. faecalis*, leading to a suggestion to consider Walls et al.'s "deferred antagonism" method. The deferred antagonism test suggests that probiotics may compete with *E. faecalis* for nutrients, potentially reducing its survival [24].

Widyarman et al.'s conclusion asserts that a concentration of 100 µg/ml of reuterin demonstrates comparable efficacy to the gold standard, 2.5% sodium hypochlorite, in the eradication of the *E. faecalis* biofilm [16]. A preceding investigation conducted by Slaughter et al. has demonstrated that probiotics possess the capacity not only to treat endodontic diseases but also to prevent their recurrence after root canal treatment [25]. Schaefer et al. highlighted reuterin's antimicrobial attributes, inducing oxidative stress in pathogens through modification of thiol groups in small molecules and proteins within cells of the pathogen and inhibiting pathogen growth [26]. Seelan et al. supported reuterin as an inhibitor of ribonucleotide reductase enzymes crucial for bacterial DNA synthesis, making it a promising endodontic irrigant [27].

Findings from Safadi et al.'s study demonstrated that the secreted products of probiotic lactic acid bacteria (LAB) strains, specifically *Lactobacillus casei* and *Lactobacillus plantarum*, exhibited notable efficacy in eliminating cells from pre-established biofilms and proficiently preventing their subsequent regrowth. This effectiveness was not replicated by the secreted products of an alternative probiotic bacterium within the same phylum, namely *Bacillus coagulans* [14].

Suissa et al. noted that probiotic strains and derivatives can alter host immune responses, protecting against pathogenic biofilms [28]. Kim et al. found that lipoteichoic acid (LTA) from *Lactobacillus plantarum* disrupted* E. faecalis* biofilm formation but showed limited efficacy in eradicating biofilm cells [29].

Limitations

Despite the encouraging outcomes associated with probiotics, there is a notable paucity of research concerning their application as intracanal irrigants. Existing studies have employed diverse probiotic species and strains, contributing to a lack of standardization in the selection protocol and administration of probiotics. Further investigations are imperative to assess the effectiveness of probiotics against additional endodontic pathogens and within a tooth-simulated environment before clinical application.

## Conclusions

Lactobacillus probiotics appear to present a safer alternative to sodium hypochlorite. While the potential efficacy of probiotics as an intracanal irrigant appears promising, the current body of research is notably limited. The existing literature predominantly explores the application of probiotics against endodontic pathogens; however, a notable deficiency exists in the standardization of the probiotic bacterial strains to be employed. Furthermore, ex vivo investigations are imperative to ascertain the specific strain, alternative probiotics, their concentration, and duration necessary for implementation before clinical interventions.
