# Correction: A Systematic Review of the Comparative Efficacy of Lactobacillus Probiotics and Sodium Hypochlorite as Intracanal Irrigants Against Enterococcus faecalis

**DOI:** 10.7759/cureus.c331

**Published:** 2025-10-01

**Authors:** Mrinalini Mrinalini, Alpa Gupta, Dax Abraham, Arun Kumar Duraisamy, Rajat Sharma

**Affiliations:** 1 Department of Conservative Dentistry and Endodontics, Manav Rachna Dental College, Faridabad, IND

This article has been corrected to fix the following errors in the PRISMA diagram: 'Records excluded after reading the title and abstract' corrected from 94 to 92.

